# Distal Intersection Syndrome Between Second and Third Dorsal Compartments of the Wrist

**DOI:** 10.7759/cureus.36919

**Published:** 2023-03-30

**Authors:** Todd H Alter, Paul V Romeo, Deidre L Bielicka, James T Monica

**Affiliations:** 1 Department of Orthopaedic Surgery, Rutgers Robert Wood Johnson Hospital, New Brunswick, USA

**Keywords:** intersection syndrome, dis, nintendinitis, dorsal extensor compartments, distal intersection syndrome

## Abstract

Distal intersection syndrome (DIS) is a rare form of tenosynovitis affecting the second and third dorsal extensor compartments of the wrist, which is rarer and more distal than the classically described intersection syndrome between the first and second compartments. In this report, we present three cases of DIS, their inciting activities, and ensuing treatment courses. Diagnosis of DIS was confirmed via MRI in all cases. Treatment modalities consisted of non-steroidal anti-inflammatory medications and varying durations of immobilization in all three patients, initially. One patient ultimately underwent surgical debridement and partial tenosynovectomy. At the end of follow-up, all patients saw a reduction in symptomatology with a return to baseline activity levels. This case report provides an overview of the possible clinical courses of DIS, as well as treatment strategies that can be implemented. Providers must maintain a high index of suspicion for this condition and treat patients with a great deal of caution, as extensor tendon rupture is possible.

## Introduction

Intersection syndrome was first described by Velpeau in 1842 as pain in the dorsal aspect of the forearm 4-8cm proximal to Lister’s tubercle of the distal radius and typically involves tenosynovitis between the first and second dorsal compartments of the wrist [1,2]. However, there have been subsequent rare reports of distal intersection syndrome (DIS), which has been defined as tenosynovitis between the second and third dorsal compartments of the wrist containing the extensor carpi radialis brevis (ECRB) and extensor carpi radialis longus (ECRL) as well as the extensor pollicis longus (EPL) tendons, respectively [3]. DIS arises where the EPL tendon traverses over the ECRB and ECRL tendons, just distal to Lister’s tubercle [4]. While the anatomic pulley created by the EPL’s course around Lister’s tubercle aids in providing leverage for thumb extension, it also predisposes the EPL tendon to injury. Furthermore, additional constriction at the level of the extensor retinaculum and the tendon’s course directly overlying the ECRB and ECRL tendons can amplify these effects [5]. Tenosynovitis subsequently develops from overuse and excessive friction within and between these dorsal compartments [4].

The most frequent presenting symptoms in DIS are pain in the dorsoradial aspect of the wrist, tenderness over Lister’s tubercle, paresthesias, and the variable presence of edema or a mass [6]. Formal diagnosis can be confirmed with ultrasound or magnetic resonance imaging (MRI), which demonstrates excessive fluid within the tendon sheaths of the second and third dorsal compartments at the level of their distal decussation [5]. Though there exists no standardized treatment algorithm, management generally begins conservatively with splinting, oral nonsteroidal anti-inflammatory drugs, and possibly a formal therapy program [7]. Steroid injection with or without lidocaine can be considered for persistent pain that does not respond to initial measures [6]. In rare circumstances, DIS has also been implicated in cases of EPL tendon rupture [4].

Currently, there is little known regarding risk factors for DIS. The patients described herein reported videogame play, cheerleading, and tennis as their primary aggravators. Certainly, tennis has been implicated in a number of tendinopathies of the upper extremity [8-10]. However, video game use has also been a suspected culprit in the development of tendonitis, even earning the nickname "Nintendinitis" to describe tendonitis of the EPL tendon at the level of the thumb due to repetitive button pushing [11]. The purpose of this case report is to describe the presentations and diagnoses of three patients, who provided informed consent, with DIS and detail their clinical courses.

## Case presentation

Case 1

Patient DM is a 29-year-old right-hand dominant male with no significant past medical history who presented with bilateral wrist pain beginning approximately four months prior to presentation. The pain was notably aggravated while playing video games, which he occasionally played for 24 hours straight, and improved when he refrained from playing. He noted swelling on the radial aspect of his wrists during flares.

On examination, the patient’s skin was intact without swelling or deformity bilaterally. At the time of examination, there was point tenderness over the left scapholunate interval and none on the right wrist. He had a full painless range of motion of his wrists and fingers. The sensation was intact to light touch, and Tinel’s and Allen’s tests were within normal limits. Radiographs of both wrists were obtained and were unremarkable. He was diagnosed with a likely dorsal ganglion cyst, given removable wrist splints, and advised to lessen his video game play.

**Figure 1:**
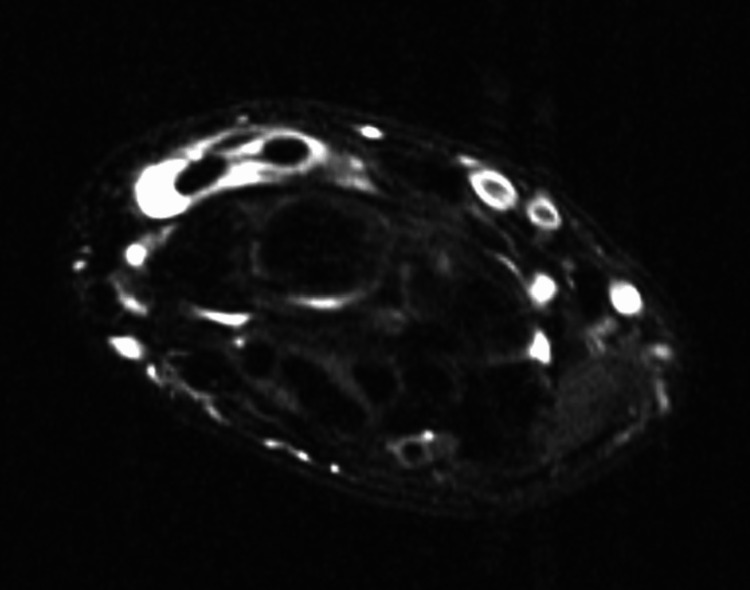
Axial T2-weighted images of the patient’s right wrist demonstrating fluid within the tendon sheaths of the ECRB/ECRL and EPL ECRB: Extensor carpi radialis brevis; ECRL: Extensor carpi radialis longus; EPL: Extensor pollicis longus

**Figure 2:**
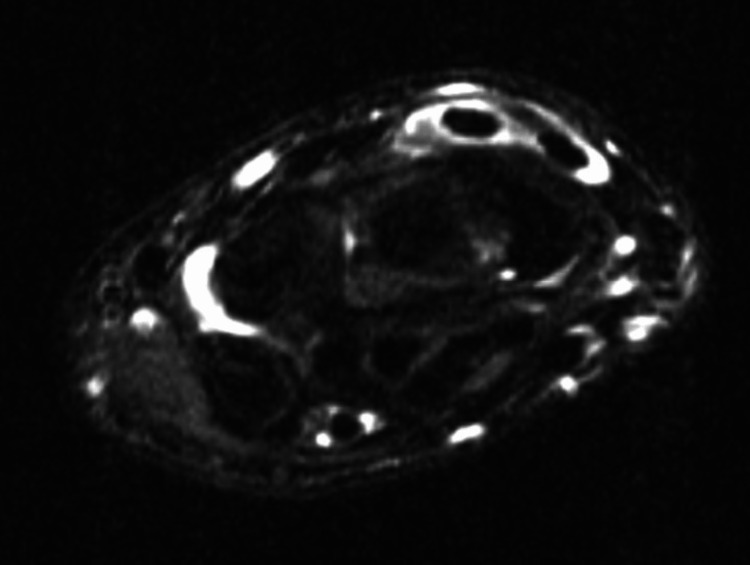
Axial T2-weighted images of the patient’s left demonstrating fluid within the tendon sheaths of the ECRB/ECRL and EPL ECRB: Extensor carpi radialis brevis; ECRL: Extensor carpi radialis longus; EPL: Extensor pollicis longus

Six months later, he returned to the office with continued symptoms despite intermittent splint wear, and he was then referred for MRIs of each wrist without contrast (Figures 1-2). This study demonstrated fluid within the tendon sheaths of the ECRB, ECRL, and EPL tendons at the level of their decussation beyond Lister’s tubercle, and the findings were remarkably similar bilaterally. These findings were consistent with the diagnosis of bilateral DIS brought on by repetitive video game play. The patient was then placed in thumb spica splints bilaterally, which were to be maintained at all times. He was given a prescription for a one-week course of an over-the-counter nonsteroidal anti-inflammatory medication. After one month of splinting, he experienced mild relief and was advised to continue splinting for another month. After the second month of splinting, the patient no longer required additional follow-up.

Case 2

Patient IA is a 14-year-old female with no past medical history who presented with right wrist pain. She participates in competitive cheerleading as a base support and tumbler and noted that her cheer activities are what most frequently bring on her symptoms. She described her pain as becoming increasingly severe over the month prior to presentation, though she has reported intermittent wrist pain for years prior to this most recent exacerbation.

On examination, the patient’s skin was intact without swelling or deformity. She had tenderness to palpation along her ECRB and ECRL tendon insertions and over her lunate. She lacked full extension, obtaining only approximately 40 degrees of passive extension compared to 80 degrees on the contralateral side. Sensation was intact to light touch, and Tinel’s and Allen’s tests were within normal limits. Radiographs of the right wrist were obtained and were unremarkable with the exception of being slightly ulnar-positive.

**Figure 3:**
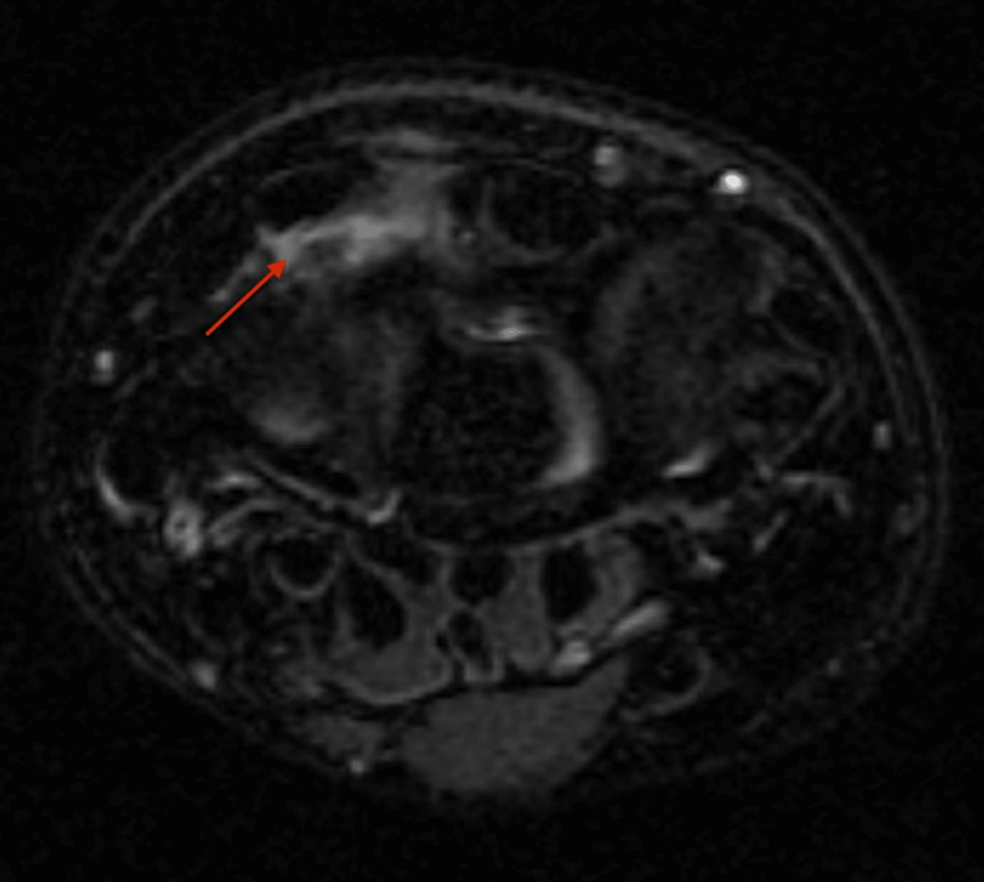
Axial T2-weighted images of the patient’s right wrist Red arrow: Peritendinous edema (external to the tendon sheath) surrounding 2nd and 3rd extensor compartments.

Due to the location of her tenderness and wrist stiffness, an MRI was obtained to evaluate for possible Kienböck's disease. However, it instead showed peritendinous edema along the distal aspects of the second and third extensor compartments with mild tendinopathy of the EPL, consistent with DIS (Figure 3). Given her cheer season was near completion, she was started on a non-steroidal anti-inflammatory medication and advised to begin rest and immobilization following the end of the season. Following this treatment course and the end of the sporting season, her symptoms resolved, and she required no further follow-up. She was advised to continue with her activities as tolerated and if her symptoms returned in the future to resume immobilization and anti-inflammatory medication. If she were to have a return of symptoms recalcitrant to conservative measures, a steroid injection could be considered.

Case 3

Patient CV is a 17-year-old male with no past medical history who presented with right wrist pain beginning approximately 3-4 years prior to presentation. At the time, he also had an associated mass and was diagnosed with a dorsal wrist ganglion cyst by another provider who aspirated the lesion and injected it with steroids. Despite gradual resolution of the mass, he continued to have pain. He noted that tennis was always the aggravating factor, but other activities including typing, writing, and video games had become bothersome if he was already experiencing a flare. Since his initial treatment, he had attempted periods of rest and exercise, as well as anti-inflammatory medications, but nothing had alleviated the pain.

**Figure 4:**
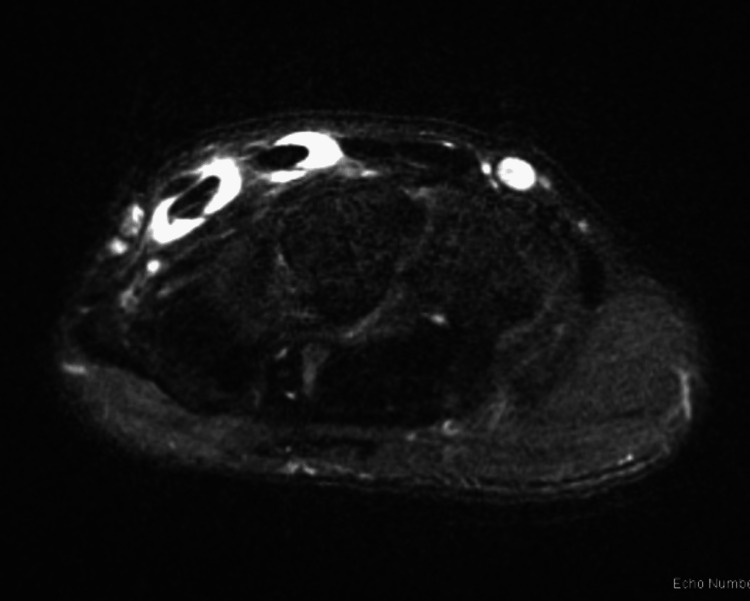
Axial T2-weighted images of the patient’s wrist demonstrating fluid within the tendon sheaths of the ECRB/ECRL and EPL ECRB: Extensor carpi radialis brevis; ECRL: Extensor carpi radialis longus; EPL: Extensor pollicis longus

On examination, the patient’s skin was intact without swelling or deformity. At the time of examination, there was point tenderness over the right scapholunate interval. He had a full painless range of motion of his wrists and fingers, with the exception of moderate pain at full wrist extension. There was no instability or pain when stressing the distal radioulnar joint. Sensation was intact to light touch, and Tinel’s and Allen’s tests were within normal limits. Radiographs of the right wrist were obtained and were unremarkable. Given the duration of his symptoms and lack of palpable mass, an MRI was obtained demonstrating tenosynovitis surrounding the second and third dorsal wrist compartments at the level of their decussation (Figure 4). In addition, a small dorsal ganglion cyst was noted emanating from the scapholunate interval.

Following the MRI findings, treatment options were discussed with the patient and his family including immobilization, aspiration and steroid injection, and surgical debridement. Due to the extended course of the patient’s symptoms and despite multiple prior conservative treatments, they elected to proceed with surgical debridement of the tenosynovitis and excision of the dorsal ganglion cyst, if present.

**Figure 5:**
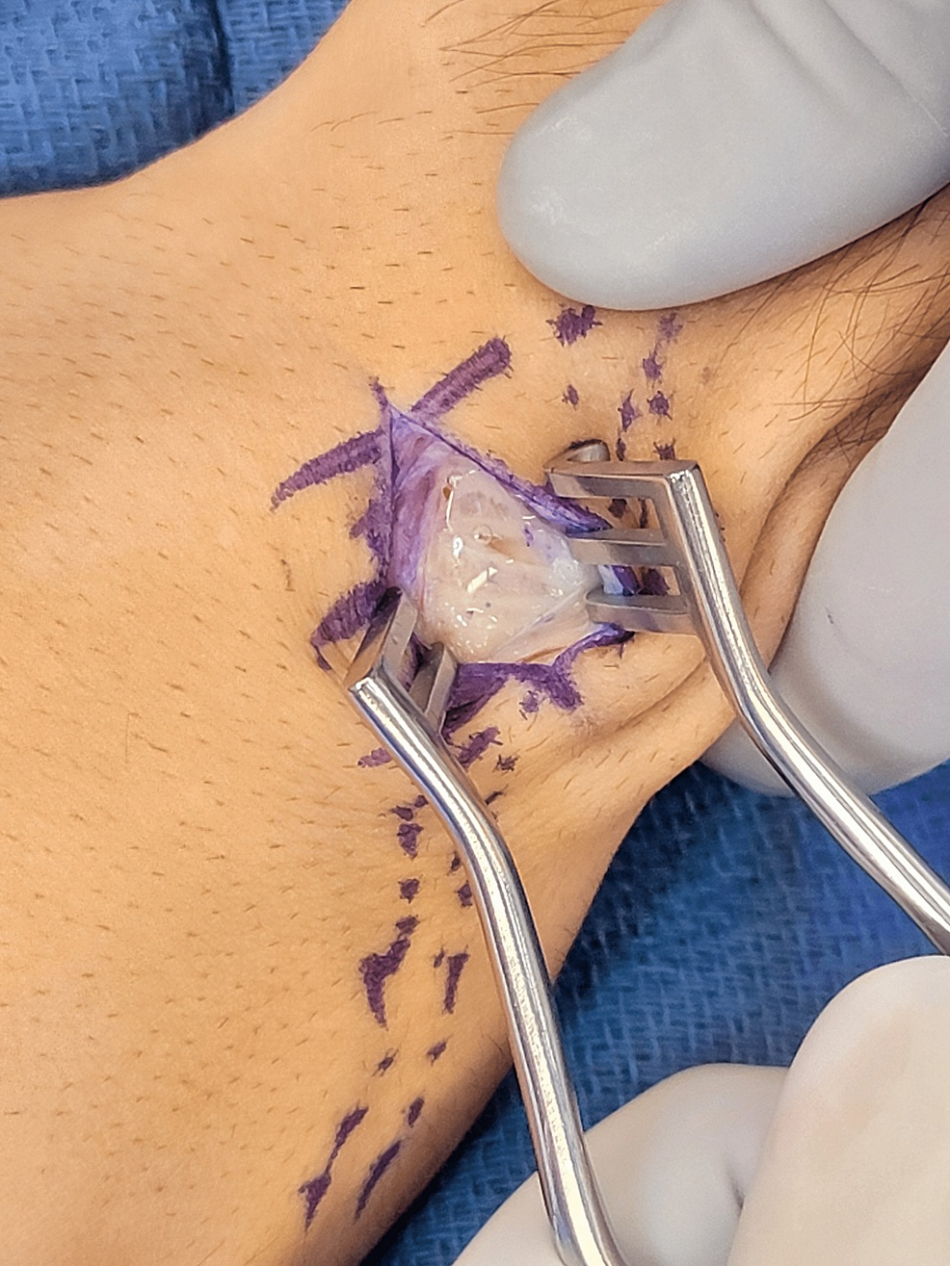
Intra-operative photograph showing a fluid collection and substantial tenosynovitis surrounding the ECRL and ECRB tendons ECRB: Extensor carpi radialis brevis; ECRL: Extensor carpi radialis longus

In the operating room, an incision centered over the intersection of the second and third extensor compartments at the base of the index and middle fingers was performed. Directly beneath the EPL tendon, a fluid collection and substantial tenosynovitis were encountered surrounding the ECRL and ECRB tendons (Figure 5). A limited tenosynovectomy was performed on the affected tissue, and the tendons were inspected and found to be intact and healthy. Attention was then focused on identifying the suspected dorsal ganglion cyst. However, no cysts were discovered, and the patient’s wound was closed. The patient’s arm was placed in a short arm cast to protect his wound and immobilize the wrist.

Post-operatively, a pathology report described the collected specimen as dense connective tissue consistent with tendon, with degenerative changes and focal lymphohistiocytic infiltrate. His cast was removed at two weeks, and physical therapy was initiated to improve his grip strength and wrist motion prior to training for the tennis season. One month postoperatively, he regained 60 degrees of active flexion and extension at the wrist with full passive motion. His strength was near baseline, and he was cleared to resume his training program.

## Discussion

DIS is a rare condition with few documented cases in the literature. Herein we describe three cases of DIS brought on by videogame play, cheerleading, and tennis. All three of these patients underwent MRI studies that demonstrated edema and tenosynovitis between the second and third dorsal compartments of the wrist. Despite similar diagnostic findings, each patient displayed differing symptom severity and subsequently underwent different treatments. Two of the patients experienced relief with immobilization and anti-inflammatory medications alone, while one ultimately underwent surgical debridement with post-operative physical therapy following failed conservative treatment.

Several authors have described their limited experiences with DIS. Parellada et al. reviewed five cases of DIS obtained via a retrospective analysis of 1,031 wrist MRIs obtained at their institution [5]. Their ages ranged from 22-78 years (mean 49 years). Two of the patients reported a history of trauma (fall from a bicycle, fall from standing). Two of the MRI studies showed a clear point of constriction at the level of the extensor retinaculum, and all studies showed varying amounts of tendinosis. Four patients recovered uneventfully with non-steroidal anti-inflammatory medications, while the fifth underwent a partial synovectomy with no recurrence at six months [5]. Interestingly, the authors withheld percutaneous injections of steroids to avoid disrupting the tenuous vascular watershed present within the EPL at the point of crossover [5].

Nam et al. discussed their experiences in three cases of DIS. The first was a 38-year-old housewife with two months of wrist pain aggravated by housework, in addition to stiffness and edema in the mornings [6]. Anti-inflammatory medication and physical therapy provided no relief, and MRI confirmed the diagnosis of DIS, which resolved after a steroid injection. She remained symptom-free at 33 months. The second patient was a 41-year-old female pianist with left wrist pain and mild edema for 3-4 years. Ultrasound confirmed DIS, and a steroid injection led to improvement but not resolution of symptoms at the two-year follow-up. The final case was a 61-year-old male office worker with right wrist pain aggravated by recreational golf for six months. Physical exam was notable only for point tenderness near Lister’s tubercle. MRI confirmed the diagnosis and steroid injection resulted in the resolution of symptoms at six months.

In a cautionary case, Mattox et al. described a 38-year-old female who presented with a painful bump on the dorsum of her wrist. Ultrasound evaluation identified tenosynovitis between the second and third dorsal compartments, the patient was diagnosed with DIS, and she was treated with immobilization [4]. However, despite seemingly appropriate diagnosis and treatment, the patient went on to develop an EPL rupture that required surgical debridement and repair. Although the pathogenesis of EPL rupture in this setting is not fully understood, it has been suggested that an effusion in an already-confined space contributes to avascular necrosis of the tendon [12]. Regardless, this case underscores the importance of early diagnosis and treatment of DIS, which could potentially prevent this rare but costly complication.

Sunagawa et al. reported on two tennis players who developed DIS with partial attritional ruptures of the ECRB tendon. The first patient, a 20-year-old male, experienced swelling and tenderness over the distal second compartment with limited wrist extension after a few weeks of tennis play [13]. Ultrasound demonstrated tenosynovitis with a partial detachment of the extensor retinaculum. Steroid injection led to the resolution of symptoms for eight months, but he then returned with an EPL rupture. He underwent a synovectomy and EPL graft with palmaris longus and was able to return to tennis three months later. The second patient, a 23-year-old male, presented with similar symptoms during his backstroke, and MRI confirmed the diagnosis of DIS. However, steroid injection and immobilization did not improve his symptoms, and a synovectomy was performed, during which attritional changes of the ECRB were noted where it contacted EPL. He too returned to tennis three months post-operatively.

While reports of DIS have increased over the last decade, it is likely that this condition is far more prevalent than previously appreciated, as it can easily be misdiagnosed. Similarly concerning is the apparently high incidence of tendinosis seen in DIS, along with multiple reports of EPL rupture. Although the three cases included in this report identify three potential etiologies for this condition, it is likely that many activities that involve repetitive movements of the hand and wrist can cause DIS. Ultimately, this report is limited in that it involves a small number of patients without standardized treatment and long-term follow-up. However, we believe it provides valuable insights into a condition that often goes unnoticed, with multiple causative agents that had not been previously reported. In the end, a thorough history and physical exam along with a high index of suspicion for the condition can aid in the proper diagnosis and treatment of these patients. Further investigations are needed to establish the best treatment algorithm and the true risk of tendon rupture.

## Conclusions

DIS is a rare condition found to be associated with repetitive movements of the hand and wrist. Clinical suspicion coupled with MRI has proved effective for accurate diagnosis. Most patients afflicted with this condition can be treated non-operatively, however, those with no relief from non-operative treatment may be considered for operative intervention. It should be noted, possible EPL rupture can develop secondary to DIS. 
